# Freely scalable and reconfigurable optical hardware for deep learning

**DOI:** 10.1038/s41598-021-82543-3

**Published:** 2021-02-04

**Authors:** Liane Bernstein, Alexander Sludds, Ryan Hamerly, Vivienne Sze, Joel Emer, Dirk Englund

**Affiliations:** 1 Research Laboratory of Electronics, Massachusetts Institute of Technology, Cambridge, MA 02139 USA; 2 NTT Research, Inc., Physics & Informatics Laboratories, Sunnyvale, CA 94085 USA; 3 Computer Science and Artificial Intelligence Laboratory, Massachusetts Institute of Technology, Cambridge, MA 02139 USA; 4 NVIDIA, Architecture Research Group, Westford, MA 01886 USA

**Keywords:** Electrical and electronic engineering, Fibre optics and optical communications, Information technology

## Abstract

As deep neural network (DNN) models grow ever-larger, they can achieve higher accuracy and solve more complex problems. This trend has been enabled by an increase in available compute power; however, efforts to continue to scale electronic processors are impeded by the costs of communication, thermal management, power delivery and clocking. To improve scalability, we propose a digital optical neural network (DONN) with intralayer optical interconnects and reconfigurable input values. The path-length-independence of optical energy consumption enables information locality between a transmitter and a large number of arbitrarily arranged receivers, which allows greater flexibility in architecture design to circumvent scaling limitations. In a proof-of-concept experiment, we demonstrate optical multicast in the classification of 500 MNIST images with a 3-layer, fully-connected network. We also analyze the energy consumption of the DONN and find that digital optical data transfer is beneficial over electronics when the spacing of computational units is on the order of $>10\,\upmu$m.

## Introduction

**Figure 1:**
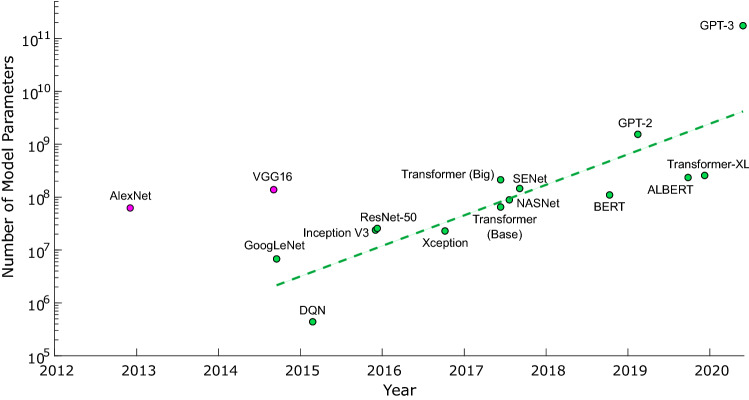
Number of parameters, i.e., weights, in recent landmark neural networks ^1,2,31–43^ (references dated by first release, e.g., on arXiv). The number of multiplications (not always reported) is not equivalent to the number of parameters, but larger models tend to require more compute power, notably in fully-connected layers. The two outlying nodes (pink) are AlexNet and VGG16, now considered over-parameterized. Subsequently, efforts have been made to reduce DNN sizes, but there remains an exponential growth in model sizes to solve increasingly complex problems with higher accuracy.

Machine learning has become ubiquitous in modern data analysis, decision-making, and optimization. A prominent subset of machine learning is the artificial deep neural network (DNN), which has revolutionized many fields, including classification^1^, translation^2^ and prediction^3,4^. An important step toward unlocking the full potential of DNNs is improving the energy consumption and speed of DNN tasks. To this end, emerging DNN-specific hardware^5–8^ optimizes data access, reuse and communication for mathematical operations: most importantly, general matrix–matrix multiplication (GEMM) and convolution^9^. However, despite these advances, a central challenge in the field is scaling hardware to keep up with exponentially-growing DNN models^10^ (see Fig. 1) due to electronic communication^11^, clocking^12^, thermal management^13^ and power delivery^14^.

To overcome these electronic limitations, optical systems have previously been proposed to perform linear algebra and data transmission. Analog weighting of optical inputs can be implemented with masks, holography or optical interference using acousto-optic modulation^15–18^, spatial light modulation^19^, electro-optic or thermo-optic modulation^20–23^, phase-change materials^24^ or printed diffractive elements^25^. Due to their analog nature, system errors can decrease the accuracy of large DNN models processed on this hardware. Prior works in *digital* optical interconnects have focused on integrated point-to-point connections^26,27^, free-space point-to-point transmission^28,29^, and small-scale free-space multicast^30^. These ideas would be difficult to scale since they incur significant overhead in number of components and introduce compounded component losses.

In this Article, we introduce a novel optical DNN accelerator that encodes inputs and weights into reconfigurable on-off optical pulses. Free-space optical elements passively transmit and copy data from memory to large-scale electronic multiplier arrays (*fan-out*). The length-independence of this optical data routing enables freely scalable systems, where single transmitters are fanned out to many arbitrarily arranged receivers with fast and energy-efficient links. This system architecture is similar to our previous coherent optical neural network^23^, but in contrast to this work and the other analog schemes described above, we propose an entirely digital system. Incoherent optical paths for data transmission (not computation) replace electrical on-chip interconnects, and can thus preserve accuracy. Unlike prior digital optical interconnect systems, our ‘digital optical neural network’ (DONN) uses free-space fan-out for data distribution to a large number of receivers for the specific application of matrix multiplication of the type found in modern DNNs.

We first illustrate the DONN architecture and discuss possible implementations. Then, in a proof-of-concept experiment, we demonstrate that digital optical transmission and fan-out with cylindrical lenses has little effect on the classification accuracy of the MNIST handwritten digit dataset (< 0.6%). Crosstalk is the primary cause of this drop in accuracy, and because it is deterministic, it can be compensated: with a simple crosstalk correction scheme, we reduce our bit error rates by two orders of magnitude. Alternatively, crosstalk can be greatly reduced through optimized optical design. Since shot and thermal noise are negligible (see “Discussion”), the accuracy of the DONN can therefore be equivalent to an all-electronic DNN accelerator.

We also compare the energy consumption of optical interconnects (including light source energy) against that of electronic interconnects over distances representative of logic, multi-chiplet interconnects and multi-chip interconnects in a 7 nm CMOS node. Multiple chips^44^ or partitioned chips^45,46^ are regularly employed to process large networks since they can ease electronic constraints and improve performance over a monolithic equivalent through greater mapping flexibility^47^, at the cost of increased communication energy. Our calculations show an advantage in data transmission costs for distances ≥ 5 $\upmu$m (roughly the size of the basic computation unit: an 8-bit multiply-and-accumulate (MAC), with length 5–8 $\upmu$m). The DONN thus scales favorably with respect to very large DNN accelerators: the DONN’s optical communication cost for an 8-bit MAC, i.e., the energy to transmit two 8-bit values, remains constant at $\sim 3$ fJ/MAC, whereas multi-chiplet systems have much higher electrical interconnect costs ($\sim 1000$ fJ/MAC), and multi-chip systems have a higher energy consumption still ($\sim 30,000$ fJ/MAC). Thus, the efficient optical data distribution provided by the DONN architecture will become critical for continued growth of DNN performance through increased model sizes and greater connectivity.

## Results

### Problem statement

**Figure 2:**
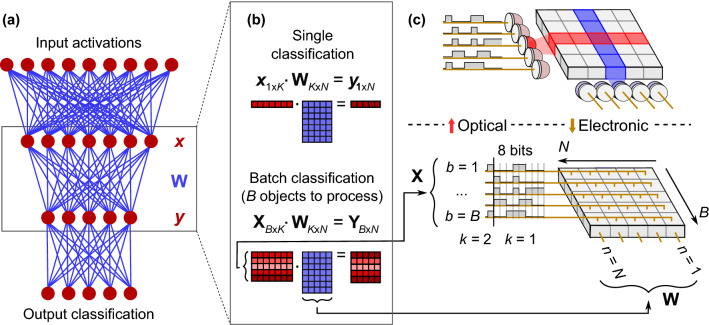
Digital fully-connected neural network (FC-NN) and hardware implementations. (**a**) FC-NN with input activations (red, vector length *K*) connected to output activations (vector length *N*) via weighted paths, i.e., weights (blue, matrix size $K\times N$). (**b**) Matrix representation of one layer of an FC-NN with *B*-sized batching. (**c**) Example bit-serial multiplier array, with output-stationary accumulation across *k*. Fan-out of **X** across $n \in \left\{ 1 \ldots N\right\}$; fan-out of **W** across $b \in \left\{ 1 \ldots B\right\}$. Bottom panel: all-electronic version with fan-out by copper wire (for clarity, fan-out of **W** not illustrated). Top panel: digital optical neural network version, where **X** and **W** are fanned out passively using optics, and transmitted to an array of photodetectors. Each pixel contains two photodetectors, where the activations and weights can be separated by, e.g., polarization or wavelength filters. Each photodetector pair is directly connected to a multiplier in close proximity.

A DNN consists of a sequence of layers, in which input activations from one layer are connected to the next layer via weighted paths (weights), as shown in Fig. 2a. We focus on inference tasks in this paper (where weights are known from prior training), which, in addition to the energy consumption problem, place stringent requirements on latency and throughput. Modern inference accelerators expend the majority of energy (> 90%) on memory access, data movement, and computation in fully-connected (FC) and convolutional (CONV) layers^5^.

Parallelized vector operations, such as matrix–matrix multiplication or successive vector–vector inner products, are the largest energy consumers in CONV and FC layers. In an FC layer, a vector ${\varvec{x}}$ of input values (‘input activations’, of length *K*) is multiplied by a matrix **W**$_{K\times N}$ of weights (Fig. 2b). This matrix–vector product yields a vector of output activations (${\varvec{y}}$, of length *N*). Most DNN accelerators process vectors in *B*-sized batches, where the inputs are represented by a matrix **X**$_{B\times K}$. The FC layer then becomes a matrix–matrix multiplication (**X**$_{B\times K}\cdot$ **W**$_{K\times N}$). CONV layers can also be processed as matrix multiplications, e.g., with a Toeplitz matrix^9^.

In matrix multiplication, fan-out, where data is read once from main memory (DRAM) and used multiple times, can greatly reduce data movement and memory access. This amortization of read cost across numerous operations is critical for overall efficiency, since retrieving a single matrix element from DRAM requires two to three orders of magnitude more energy than the MAC^11^. A simple input-weight product illustrates the benefit of fan-out, since activation and weight elements appear repeatedly, as highlighted by the repetition of $X_{11}$ and $W_{11}$:1

Consequently, DNN hardware design focuses on optimizing data transfer and input and weight matrix element reuse. Accelerators based on conventional electronics use efficient memory hierarchies, a large array of tightly packed processing elements (PEs, i.e., multipliers with or without local storage), or some combination of the these approaches. Memory hierarchies optimize temporal data reuse in memory blocks near the PEs to boost performance under the constraint of chip area^9^. This strategy can enable high throughput in CONV layers^5^. With fewer intermediate memory levels, a larger array of PEs (e.g., TPU v1^8^) can further increase throughput and lower energy consumption on workloads with a high-utilization mapping due to potentially reduced overall memory accesses and a greater number of parallel multipliers (spatial reuse). Therefore, for workloads with large-scale matrix multiplication such as those mentioned in the Introduction, if we maximize the number of available PEs, we can improve efficiency.

### Digital optical neural network architecture

Our DONN architecture replaces electrical interconnects with optical links to relax the design constraints of reducing inter-multiplier spacing or colocating multipliers with memory. Specifically, optical elements transfer and fan out activation and weight bits to electronic multipliers to reduce communication costs in matrix multiplication, where each element $X_{bk}$ is fanned out *N* times, and $W_{kn}$ is fanned out *B* times. The DONN scheme shown in Fig. 2c spatially encodes the first column of **X**$_{B\times K}$ activations into a column of on-off optical pulses. At the first time step, the activation matrix transmitters fan out the first bit of each of the matrix elements $X_{b1}, \forall b \in \left\{ 1 \ldots B\right\}$ to the PEs (here, $k=1$). Simultaneously, a row of weight matrix light sources transmits the corresponding weight bits $W_{1n}$ to each PE. The photons from these activation and weight bits generate photoelectrons in the detectors, producing the voltages required at the inputs of electronic multipliers (either 0 V for a ‘0’ or 0.8 V for a ‘1’). After 8 time steps, a multiplier has received $2\times 8$ bits (8 bits for the activation value and 8 bits for the weight value), and the electronic multiplication occurs as it would in an all-electronic system. The activation-weight product is completed, and is added to the locally stored partial sum. The entire matrix–matrix product is therefore computed in $8\times K$ time steps; this dataflow is commonly called ‘output stationary’. Instead of this bit-serial implementation, bits can be encoded spatially, using a bus of parallel transmitters and receivers. The trade-off between added energy and latency in bit-serial multiplication versus increased area from photodetectors for a parallel multiplier can be analyzed for specific applications and CMOS nodes.

**Figure 3:**
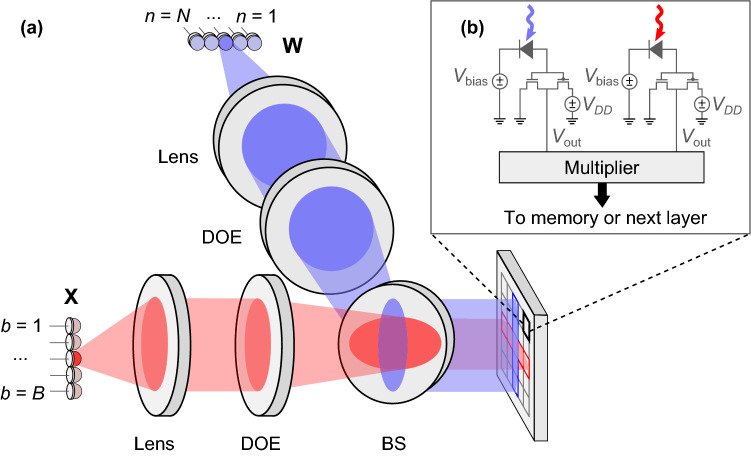
Possible implementation of digital optical neural network. (**a**) Digital inputs and weights are transmitted electronically to an array of light sources (red and blue, respectively, illustrating different paths). Single-mode light from a source is collimated by a spherical lens (Lens), then focused to a 1D spot array by a diffractive optical element (DOE). A 50:50 beamsplitter brings light from the inputs and weights into close proximity on a custom CMOS receiver. (**b**) Example circuit with 2 photodetectors (biased by voltage $V_\text {bias}$) per PE: 1 for activations; 1 for weights. Received bits ($V_\text {out}$) proceed to multiplier, then memory or next layer.

We illustrate an exemplary experimental DONN implementation in Fig. 3. Each source in a linear array of vertical cavity surface emitting lasers (VCSELs) or $\upmu$LEDs emits a cone of light into free space, which is collimated by a spherical lens. A diffractive optical element (DOE) focuses the light to a 1D spot array on a 2D receiver, where the activations and weights are brought into close proximity using a beamsplitter. ‘Receiverless’ photodetectors^48^ convert the optical signals to the electrical domain. An electronic multiplier then multiplies the values. The output is either saved to memory, or routed directly to another DONN that implements the next layer of computation. Note that the data distribution pattern is not confined to regular rows and columns. A spatial light modulator (SLM), an array of micromirrors, scattering waveguides or a DOE can route and fan out bits to arbitrary locations. Since free-space propagation is lossless and mirrors, SLMs and diffractive elements are highly efficient (> 95%), most length- or receiver-number-dependent losses can be attributed to imperfect focusing, e.g., from optical aberrations far from the optical axis. These effects can be mitigated through judicious optical design. We assume for the remainder of our analysis that energy is length-independent.

### Bit error rate and inference experiments

**Figure 4:**
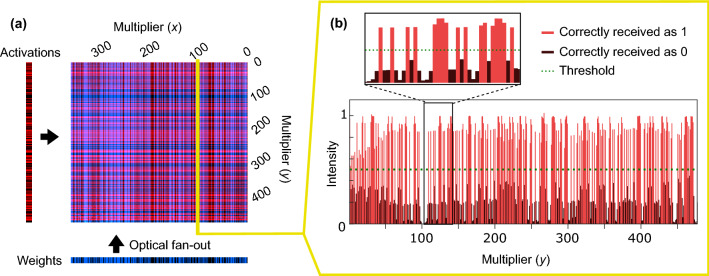
Background-subtracted and normalized receiver output from free-space digital optical neural network experiment with random vectors of ‘1’s and ‘0’s displayed on DMDs. (**a**) Full 2D image. (**b**) One column: pixels received as ‘1’ in red and ‘0’ in black.

We used a DONN implementation similar to Fig. 3a to test optical digital data transmission and fan-out for DNNs, as described in “Methods”. In our first experiment, we determined the bit error rate of our system. Figure 4a shows an example of a background-subtracted and normalized image, captured on the camera when the digital micromirror devices (DMDs) displayed random vectors of ‘1’s and ‘0’s. The camera’s de-Bayering algorithm (described in “Methods”), as well as optical aberrations and misalignment, caused some crosstalk between pixels (see Fig. 4b). Using a region of $357\times 477$ superpixels on the camera, we calculated bit error rates (in a single shot) of $1.2\times 10^{-2}$ and $2.6\times 10^{-4}$ for the blue and red channels, respectively. When we confined the region of interest to $151\times 191$ superpixels, the bit error rate (averaged over 100 different trials, i.e., 100 pairs of input vectors) was $4.4\times 10^{-3}$ and $4.6\times 10^{-5}$ for the blue and red arms. See Supplementary Note 1 for more details on bit error rate and error maps. Because crosstalk is deterministic, and not a source of random noise, we can compensate for it. We applied a simple crosstalk correction scheme that assumes uniform crosstalk on the detector and subtracts a fixed fraction of an element’s nearest neighbors from the element itself (see Supplementary Note 2). The bit error rates for the blue and red channels then respectively dropped to $2.9\times 10^{-3}$ and 0 for the $357\times 477$-pixel, single shot image and $2.6\times 10^{-5}$ and 0 for the $151\times 191$-pixel, 100-image average. In other words, after crosstalk correction, there were no errors in the red channel, and the errors in the blue channel dropped significantly.

**Figure 5:**
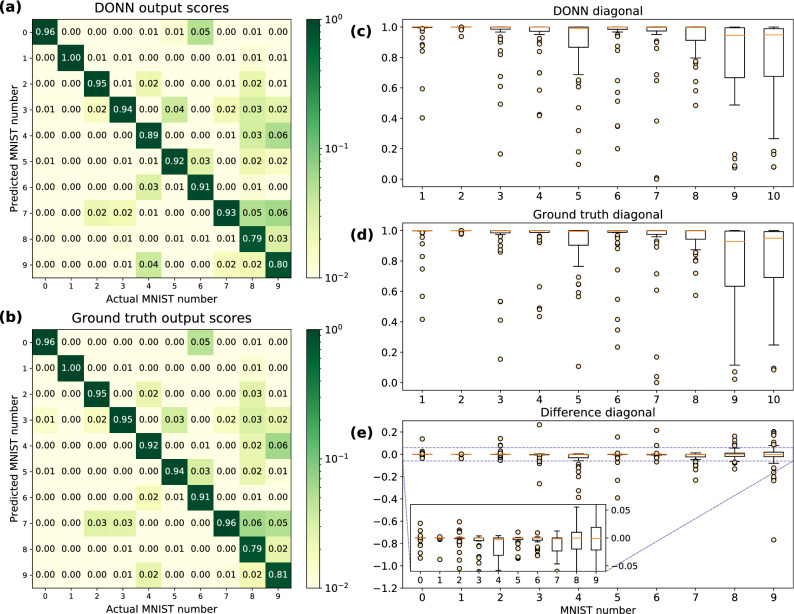
Experimentally measured 3-layer FC-NN output scores, otherwise known as confusion matrix, for 500 MNIST images from test dataset. The values along the diagonal represent correct classification by the model. Each column is an average of $\sim 50$ vectors. (**a**) DONN output scores (no crosstalk correction applied). (**b**) Ground-truth (all-electronic) output scores. (**c**, **d**) Box plot of the diagonals of subfigures (**a**) and (**b**) respectively. (**e**) Difference in diagonals of DONN output scores versus ground-truth output scores. Box plots represent the median (orange), interquartile range (IQR, box) and ‘whiskers’ extending 1.5 IQRs beyond the first and third quartile; outliers are displayed as yellow circles.

Next, we experimentally tested the DONN’s effect on the classification accuracy of 500 MNIST images using a three-layer (i.e., two-hidden-layer), fully-connected neural network (FC-NN), with the dataset and training steps described in Supplementary Note 3. We compared our uncorrected experimental classification results with inference performed entirely on CPU (ground truth) in two ways. The simplest analysis, reported in Table 1, shows a 0.6% drop in classification accuracy for the DONN versus the ground truth values (or 3 additional incorrectly classified images). Figure 5 illustrates more detailed results, where we analyzed the network output scores. An output score is roughly equivalent to the assigned likelihood that an input image belongs to a given class, and is defined as the normalized (via the softmax function) output vector of a DNN. We found that, along the matrix diagonal, the first and third quartiles in the difference in output scores between the DONN and the ground truth have a magnitude < 3%. The absolute difference in average output scores is also < 3%. We also performed this experiment with a single hidden layer (‘2-layer’ case), and achieved similar results (a 0.4% drop in accuracy, or 2 misclassified images). No crosstalk error correction was applied to these results to illustrate the worst-case impact on accuracy.

**Table 1:** MNIST classification accuracy of DONN (no crosstalk correction applied) versus all-electronic hardware with custom fully-connected neural network models.

	2 layers (%)	3 layers (%)
Electronic (ground truth)	95.8	96.4
DONN	95.4	95.8

### Energy analysis: DONN compared with all-electronic hardware

**Figure 6:**
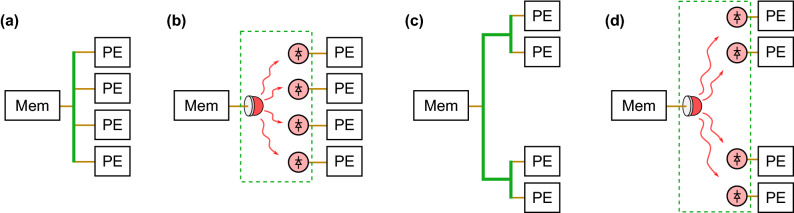
Fan-out of one bit from memory (Mem) to multiple processing elements (PEs). (**a**) Fan-out by electrical wire to a row of PEs in a monolithic chip. (**b**) DONN equivalent of monolithic chip, where green wire is replaced by optical paths. (**c**) Fan-out by electrical wire to blocks of PEs divided into chiplets, or separated by memory and logic. (**d**) DONN equivalent of fan-out to PEs in multiple blocks [energetically equivalent to (**b**)].

In this section, we compare the theoretical interconnect energy consumption of the DONN with its all-electronic equivalent, where interconnects are illustrated in green in Fig. 6. We assume an implementation in a 7 nm CMOS process for both cases. The interconnect energy, which must include any source inefficiencies, is the energy required to charge the parasitic wire, detector, and inverter capacitances, where a CMOS inverter is representative of the input to a multiplier. See “Methods” for full energy calculations. In the electronic case, a long wire transports data to a row of multipliers using low-cost (0.06 fJ/bit) repeaters (see Supplementary Note 6). The wire has a large parasitic capacitance, but also produces an effective electrical fan-out. In the DONN, the energetic requirements of the detectors contrast with those of conventional optical receivers, which aim to maximize sensitivity to the optical input field, rather than minimize the energetic cost of the system as a whole. The parameters used for electronic and optical components are summarized in Table 2, where $h\nu /e$ must be greater than or equal to the bandgap $E_\text {g}$ of the detector material (here, we have chosen silicon as an example, and set $h\nu /e = E_\text {g}$). $C_{\text {wire}}/\upmu \text {m}$ is the wire capacitance per micrometer, $V_{DD}$ is the supply voltage and $C_\text {det}$ is a theoretical approximation of the capacitance of a receiverless cubic photodetector^48^ with surface area $A_\text {det} = (1\times 1)\,\upmu \text {m}^2$. Several past examples of small CMOS integrated detectors in older CMOS nodes^49,50^ showcase the feasibility of receiverless detectors in advanced nodes. The optical source power conversion efficiency (wall-plug efficiency, i.e., WPE) is a measured value for VCSELs^51,52^. $C_\text {T}$ is an approximation for the capacitance of an inverter^48,53^. $L_\text {wire}$ is the distance between MAC units in various scenarios: with abutted MAC units (intra-chiplet), between chiplets (inter-chiplet) and between chips (inter-chip).

**Figure 7:**
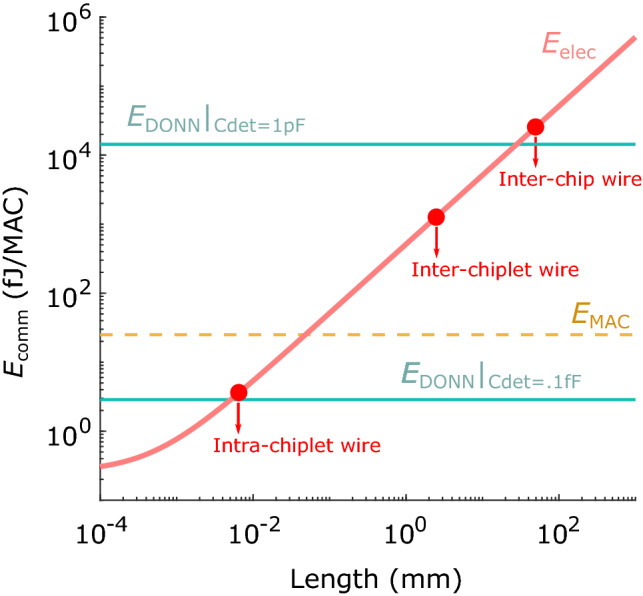
Energy required to transmit 16 bits (communication energy per 8-bit MAC, i.e., $E_\text {comm}$). Electronic data transfer energy ($E_\text {elec}$) increases with wire length, whereas optical data transfer energy ($E_\text {DONN}$) remains constant. Optical data transfer evaluated for two detector capacitances: $C_\text {det}=1$ pF for large, commercially-available photodiodes^63^; and $C_\text {det}=0.1$ fF for emerging receiverless, (1 $\upmu$m)$^3$-sized cubic detectors in modern CMOS processes^48^. Below $C_\text {det}=0.1$ fF, the capacitance of the overall receiver becomes limited by the capacitance of the CMOS inverter. Otherwise, the capacitance of the photodetector is energy-limiting. Energy of one 8-bit multiply-and-accumulate operation ($E_\text {MAC} = 25$ fJ/MAC) also shown for reference.

As shown in Fig. 7, we find that the optical communication energy is $E_\text {comm} \approx 3$ fJ/MAC, independent of length, when we use receiverless detectors in a modern CMOS process (limited by the photodetector and inverter capacitances). On the other hand, the electrical interconnect energy scales from $E_\text {comm} = 3$–4 fJ/MAC for inter-multiplier communication for abutted MAC units, to $\sim$1000 fJ/MAC for inter-chiplet interconnects, to $\sim$30,000 fJ/MAC for inter-chip interconnects. The crossover point where the optical interconnect energy drops below the electrical energy occurs when $L_{\text {wire}} \ge 5\,\upmu \text {m}$. The DONN therefore provides an improvement in the interconnect energy for data transmission and can scale to greatly decrease the energy consumption of data distribution with regular distribution patterns. In Fig. 7, we have also included the optical communication energy per MAC with a large, commercial photodiode, which illustrates the need for receiverless photodetectors in a 7 nm CMOS process. In the future, plasmonic photodetectors may lower the capacitance further than 0.1 fF^54^.

**Table 2:** Parameters.

$C_{\text {wire}}/\upmu \text {m}$	$\sim$0.2 fF$/\upmu$m^48,55,56^
$C_{\text {T}}$	$\sim$0.1 fF^48,53^
$C_\text {det}$	0.1 fF^48^
$h \nu / e$	1.12 eV
WPE	$\sim$0.5^51,52^
$A_\text {det}$	$1\,\upmu \text {m} \times 1 \,\upmu \text {m}$^48^
$L_{\text {wire}\_\text {intra-chiplet}}$	5-8 $\upmu$ $\hbox {m}^\dagger$
$L_{\text {wire}\_\text {inter-chiplet}}$	2.5 mm^45^
$L_{\text {wire}\_\text {inter-chip}}$	$\sim$5 cm^57^
$V_{DD}$	0.80 V^58^
$E_\text {MAC}$*	25 fJ/MAC^11,58^

$^\dagger$We assume a square multiplier and scale reported 8-bit multiplier areas in a 45 nm node^59–61^ to a 7 nm node (the current state of the art) with the scaling factors from literature^58^. A MAC unit comprises both an 8-bit multiplier and a 32-bit adder, so we are placing a lower bound on the minimum length of $L_\text {wire}$. Recent work^62^ optimizes MAC units for DNNs, and reports a $337\,\upmu \text {m}^2$ area in a 28 nm node, where the MAC unit comprises an 8-bit multiplier and a 32-bit adder. Extrapolated to a 7 nm node with a fourth-order polynomial fit of the scaling factors from literature^58^, the MAC unit is of size ($7\,\upmu \text {m})^2$, which falls within the 5-8 $\upmu$m range.

*$E_\text {MAC}$, the energy required for one multiply-and-accumulate, shown for reference.

## Discussion

With minimal impact on accuracy, the DONN yields an energy advantage over all-electronic accelerators with long wire lengths for digital data transfer. In our proof-of-concept experiment, we performed inference on 500 MNIST images with 2- and 3-layer FC-NNs and found a < 0.6% drop in accuracy and a < 3% absolute difference in average output scores with respect to the ground truth implementation on CPU. We attributed these errors to crosstalk due to imperfect alignment and blurring from the camera’s Bayer filter. In fact, a simple crosstalk correction scheme lowered measured bit error rates by two orders of magnitude. We could thus transmit bits with 100% measured fidelity in the activation arm (better aligned than the weight arm), which illustrates that crosstalk can be mitigated and possibly eliminated through post-processing, charge sharing at the detectors, greater spacing of receivers, or optimized design of optical elements and receiver pixels. In the hypothetical regime where error due to crosstalk is negligible, the remaining noise sources are shot and thermal noise. Intuitively, shot and thermal noise are also present in an all-electronic system, and the number of photoelectrons at the input to an inverter in the DONN is equal to the number of electrons at the input to an inverter in electronics. Therefore, if these noise sources do not limit accuracy in the all-electronic case, the same can be said for the DONN^48^. For mathematical validation that shot and thermal noise have a trivial impact on bit error rate in the DONN, see Supplementary Note 7. These analyses demonstrate that the fundamental limit to the accuracy of the DONN is no different than the accuracy of electronics, and thus, we do not expect accuracy to hinder DONN scaling in an optimized system.

In our theoretical energy calculations, we compared the length-independent data delivery costs of the DONN with those of an all-electronic system. We found that in the worst case, when multipliers are abutted in a multiplier array, optical transmitters have a similar interconnect energy cost compared to copper wires in a 7 nm node. The regime where the DONN shows important gains over copper interconnects is in architectures with increased spacing between computation units. As problems scale beyond the capabilities of existing single electronic chips, multiple chiplets or chips perform DNN tasks in concert. In the multi-chiplet and multi-chip cases, the costs to transmit two 8-bit values in electronics ($\sim$1000 fJ/MAC and $\sim$30,000 fJ/MAC, respectively) are therefore significantly larger than that of an 8-bit MAC (25 fJ/MAC)^11,58^. On the other hand, in optics, the interconnect cost ($\sim$3 fJ/MAC, including source energy) remains an order of magnitude smaller than the MAC cost. Since multi-chiplet and multi-chip systems offer a promising approach to increasing throughput on large DNN models, optical connectivity can further these scaling efforts by reducing inter-chiplet and inter-chip communication energy by orders of magnitude. We further discuss the scalability of the DONN in Supplementary Note 8. In terms of the DONN’s area, we assume the added chip area at the receiver is negligible, since the area of a photodetector $A_\text {det}=1\,\upmu \text {m}^2$ is $\sim$50$\times$ smaller than a MAC unit of size $(L_{\text {wire}\_\text {intra-chiplet}})^2$. Furthermore, for many practical applications (e.g., workstations, servers, data centers), chip area, which sets fabrication cost, and energy efficiency are much more important than overall packaged volume. In data centers today, space is required between chips for heat sinks and airflow, and the addition of lenses need not increase this volume significantly. Finally, as discussed in Supplementary Note 9, optical devices do not restrict the clock speed of the system since their bandwidths are $>10$ GHz. In fact, the clock speed of a digital electronic system is generally limited to $\sim 1$ GHz due to thermal dissipation requirements; it could be improved in the DONN, since greater component spacing for thermal management would not increase energy consumption.

Because length-independent data distribution is a tool currently unavailable to digital system designers, relaxing electronic constraints on locality can open new avenues for DNN accelerator architectures. For example, memory can be devised such that numerous small pieces of memory are located far away from the point of computation and reused many times spatially, with a small fixed cost for doing so. Designers can then lay out smaller memory blocks with higher bandwidth, lower energy consumption, and higher yield. If memory and computation are spatially distinct, we have the added benefit of allowing for more compact memories that consume less energy and area, e.g., DRAM, which is fabricated with a different process than typical CMOS to achieve higher density than on-chip memories. Furthermore, due to its massive fan-out potential, the DONN can, firstly, reduce overhead by minimizing a system’s reliance on a memory hierarchy and, secondly, amortize the cost of weight delivery to multiple clients running the same neural network inference on different inputs. Additionally, some newer neural network models require irregular connectivity (e.g., graph neural networks, which show state-of-the-art performance on recommender systems, but are restricted in size due to insufficient compute power^64,65^). These systems have arbitrary connections with potentially long wire lengths between MAC units, representing different edges in the graph. The DONN can implement these links without incurring additional costs in energy from a complex network-on-chip in electronics. Yet another instance of greater distance between multipliers is in higher-bit-precision applications, as in training, which require larger MAC units.

In future work, we plan to assess the performance of the DONN on state-of-the-art DNN workloads, such as the models described in MLPerf^66^. Firstly, we will benchmark the DONN against all-electronic state-of-the-art accelerators by using Timeloop^67^. Through a search for optimal mappings (ways to organize data and computation), this software can simulate the total energy consumption and latency of running various workloads on a given hardware architecture, including computation and memory access. Timeloop therefore enables us to perform an in-depth comparison of all-electronic accelerators against the proposed instances of the DONN, including variable data transmission costs for different electronic wire lengths. Second, we will design an optical setup and receiver to reduce experimental crosstalk, power consumption and latency. We can then test larger workloads on this optimized hardware. Finally, beyond neural networks, there are many examples of matrix multiplication which a DONN-style architecture can accelerate, such as optimization, Ising machines and statistical analysis, and we plan to investigate these applications as well.

In summary, the DONN implements arbitrary transmission and fan-out of data with an energy cost per MAC that is independent of data transmission length and number of receivers. This property is key to scaling deep neural network accelerators, where increasing the number of processing elements for greater throughput in all-electronic hardware typically implies higher data communication costs due to longer electronic path length. Contrary to other proposed optical neural networks^21–25^, the DONN does not require digital-to-analog conversion and is therefore less prone to error propagation. The DONN is also reconfigurable, in that the weights and activations can be easily updated. Our work indicates that the length-independent communication enabled by optics is useful for digital neural network system design, for example to simplify memory access to weight data. We find that optical data transfer begins to save energy when the spacing of MAC computational units is on the order of > 10 μm. More broadly, further gains can be expected through the relaxation of electronic system architecture constraints.

## Methods

### Digital optical neural network implementation for bit error rate and inference experiments

We performed bit error rate and inference experiments with optical data transfer and fan-out of point sources using cylindrical lenses. Two digital micromirror devices (DMDs, Texas Instruments DLP3000, DLP4500) illuminated by spatially-filtered and collimated LEDs (Thorlabs M625L3, M455L3) acted as stand-ins for the two linear source arrays. For the input activations/weights, each 10.8 $\upmu$m-long mirror in one DMD column/row either reflected the red/blue light toward the detector (‘1’) or a beam dump (‘0’). Then, for each of the DMDs, an $f=100\,\text {mm}$ spherical lens followed by an $f=100\,\text {mm}$ cylindrical achromatic lens imaged one DMD pixel to an entire row/column of superpixels of a color camera (Thorlabs DCC3240C). Each camera superpixel is made up of four pixels of size (5.3 $\upmu$m)^2^: two green, one red and one blue. The camera acquisition program applies a ‘de-Bayering’ interpolation to automatically extract color information for each sub-pixel; this interpolation causes blurring, and therefore it increases crosstalk in our system. In a future version of the DONN, a specialized receiver will reduce this crosstalk and also operate at a higher speed.

To process the image received on the camera, we subtracted the background, normalized, then thresholded by a fixed value for each channel. (We acquired normalization and background curves with all DMD pixels in the ‘on’ and ‘off’ states, respectively. This background subtraction and normalization could be implemented on-chip by precharacterizing the system, and biasing each receiver pixel by some fixed voltage.) If the detected intensity was above the threshold value, it was labeled a ‘1’; below threshold, a ‘0’. For the bit error rate experiments, we compared the parsed values from the camera with the known values transmitted by the DMDs, and defined the bit error rate as the number of incorrectly received bits divided by the total number of bits. In the inference experiments, the DMDs displayed the activations and pre-trained weights, which propagated through the optical system to the camera. After background subtraction and normalization, the CPU multiplied each activation with each weight, and applied the nonlinear function (ReLU after the hidden layers and softmax at the output). We did not correct for crosstalk here, to illustrate the worst-case scenario of impact on accuracy. The CPU then fed the outputs back to the input activation DMD for the next layer of computation. We used a DNN model with two hidden layers with 100 activations each and a 10-activation output layer. We also tested a model with a single hidden layer with 100 activations.

### MNIST preprocessing

For the inputs to the network, a bilinear interpolation algorithm transformed the $28\times 28$-pixel images into $7\times 7$-pixel images, which were then flattened into a 1D 49-element vector. The following standard mapping quantized both input and weight matrices into 8-bit integer representations:

2 $$\begin{aligned} {\mathrm{Quantized}} = {\mathrm{QuantizedMin}} + \frac{({\mathrm{Input}} - {\mathrm{Floating Min}})}{{\mathrm{Scale}}} \end{aligned}$$

where Quantized is the returned value, QuantizedMin is the minimum value expressible in the quantized datatype (here, always 0), Input is the input data to be quantized, FloatingMin is the minimum value in Input, and Scale is the scaling factor to map between the two datatype ranges $\left( \frac{{\mathrm{FloatingMax}} - {\mathrm{FloatingMin}}}{{\mathrm{QuantizedMax}} - {\mathrm{QuantizedMin}}}\right)$. See gemmlowp documentation^68^ for more information on implementations of this quantization. In practice, 8-bit representations are widely used in DNNs, since 8-bit MACs are generally sufficient to maintain accuracy in inference^8,69,70^.

### Electronic and optical interconnect energy calculations

When an electronic wire transports data over a distance $L_\text {wire}$ to the gate of a CMOS inverter (representative of a full-adder’s input, the basic building block of multipliers), the energy consumption per bit is:

3 $$\begin{aligned} E_\text {elec}/\text {bit} = \tfrac{1}{4}\left( \tfrac{C_{\text {wire}}}{\upmu \text {m}} \cdot L_{\text {wire}}+C_\text {T}\right) \cdot V_{DD}^2 \end{aligned}$$

where $V_{DD}$ is the supply voltage, $C_{\text {wire}}/\upmu \text {m}$ is the wire capacitance per micrometer, $L_{\text {wire}}$ is the wire length between two multipliers and $C_\text {T}$ is the inverter capacitance. Interconnects consume energy predominantly when a load capacitance, such as a wire, is charged from a low (0 V) to a high ($\sim$1 V) voltage, i.e., in a $0\rightarrow 1$ transition. If we assume a low leakage current, maintaining a value of ‘1’ (i.e., $1\rightarrow 1$) consumes little additional energy. To switch a wire from a ‘1’ to a ‘0’, the wire is discharged to the ground for free (Supplementary Note 4). Lastly, maintaining a value of ‘0’ simply keeps the voltage at 0 V, at no cost. Assuming a random distribution of ‘0’ and ‘1’ bits, we therefore include a factor of 1/4 in Eq. (3) to account for this dependence on switching activity.

In the DONN, a light source replaces the wire for fan-out. The low capacitances of the receiverless detectors in the DONN allow for the removal of receiving amplifiers^48^. Thus, the DONN’s minimum energy consumption corresponds to the optical energy required to generate a voltage swing of 0.8 V on the load capacitance (i.e., the photodetector ($C_\text {det}$) and an inverter ($C_\text {T}$)), all divided by the source’s power conversion efficiency (wall-plug efficiency, WPE). Subsequent transistors in the multiplier are powered by the off-chip voltage supply, as in the all-electronic architecture. Assuming a detector responsivity of $\sim$1^71^, the DONN interconnect energy cost is:

4 $$\begin{aligned} E_\text {DONN}/\text {bit} = \tfrac{1}{2\cdot \text {WPE}}\cdot h\nu \cdot n_\text {p} \end{aligned}$$

where $h\nu$ is the photon energy and the number of photons per bit, $n_\text {p}$, is determined by:

5 $$\begin{aligned} n_\text {p} =\frac{\left( C_{\text {det}}+C_\text {T}\right) \cdot V_{DD}}{e} \end{aligned}$$

As in the all-electronic case, we assume low leakage on the receiverless photodetector. Photons are received for every ‘1’ and therefore, to avoid charge buildup, charge on the output capacitor must be reset after every clock cycle. In Supplementary Note 5, we propose a CMOS discharge circuit that actively resets the receiver. (Another possible method is a dual-rail encoding scheme^48^.) Thus, the switching activity factor is 1/2 instead of 1/4: as for the all-electronic case, we assume a random distribution of bits, but here, both $1\rightarrow 1$ and $0\rightarrow 1$ have a nonzero cost.

The energy consumption per 8-bit multiply-and-accumulate ($E_\text {comm}$ in fJ/MAC) is simply the energy per bit multiplied by 16, representative of transmitting two 8-bit values.

## Supplementary Information

Supplementary Information 1.

## Data Availability

The data generated and analyzed in this study are available from the corresponding authors upon reasonable request.

## Code availability

Code used for acquiring and processing the MNIST dataset can be found at https://github.com/alexsludds/Digital-Optical-Neural-Network-Code. Code used for image processing, hardware control, and calculations for energy, crosstalk and bit error rate is available from the corresponding authors upon reasonable request.
