# Supplementary material for: Freely scalable and reconfigurable optical hardware for deep learning
Source: Sci Rep. 2021 Feb 4;11:3144. doi: 10.1038/s41598-021-82543-3 (PMC7862662; doi:10.1038/s41598-021-82543-3)
Supplement: Supplementary file 1 — Supplementary Information 1. [file 41598_2021_82543_MOESM1_ESM.pdf]

Freely scalable and reconfigurable optical hardware for deep learning: supplementary material

Liane Bernstein[1,+,*], Alexander Sludds[1,+,*], Ryan Hamerly[1,2], Vivienne Sze[1], Joel Emer[3,4], and Dirk Englund[1,*]

[1]Research Laboratory of Electronics, Massachusetts Institute of Technology, Cambridge, MA 02139, USA
[2]NTT Research, Inc., Physics & Informatics Laboratories, Sunnyvale, CA 94085, USA
[3]Computer Science and Artificial Intelligence Laboratory, Massachusetts Institute of Technology, Cambridge, MA 02139, USA
[4]NVIDIA, Architecture Research Group, Westford, MA 01886, USA
[*]lbern@mit.edu, asludds@mit.edu, englund@mit.edu
[+]These authors contributed equally to this work

Supplementary Note 1: Bit error rate due to crosstalk

Here, we show experimental bit error rate maps for both the blue and red channels. Each DMD pixel is fanned out to a row (column) of superpixels on the camera for the input activations (weights). The Bayer filter allows the discrimination of the input activations from the weights into red and blue channels, respectively. Since the camera has four sub-pixels per superpixel, we bin the sub-pixels into 2×2 blocks. As described and shown in Fig. 4 of the main text, random vectors of '1's and '0's were displayed on the DMDs to assess bit error rates in data transmission from two 1D source arrays to the camera. In Fig. S1, we show bit error rate maps. Figures S1(a) and (c) show the error from a single shot (one random vector pair displayed on the DMDs). Figures S1(b) and (d) show the error averaged over 100 frames (100 different random vector pairs displayed on the DMDs) in the low-error region of interest used for the proof-of-concept experiment. The error is larger on the edges of each image due to optical aberrations, and is larger in the blue channel than the red channel due to misalignment.

Supplementary Note 2: Crosstalk correction

The bit error rate described in the previous section is mainly attributable to optical crosstalk at the detector due to imperfect lenses and alignment. Since this error is deterministic (as opposed to random fluctuations), it can be compensated by post-processing. To illustrate this principle, we performed simple crosstalk correction: we multiplied each line of an image detected on the camera by a tridiagonal crosstalk reduction matrix, per equation (1) (where $\overline{I_{:n}}$ is the corrected line of the camera image).

$$\begin{bmatrix} \overline{I_{1n}} \\ \overline{I_{2n}} \\ \vdots \\ \\ \end{bmatrix} = \begin{bmatrix} 1 & -\xi & 0 & & \\ -\xi & 1 & -\xi & & \\ 0 & -\xi & 1 & \ddots & \\ & & \ddots & \ddots & \\ & & & & 1 \end{bmatrix} \begin{bmatrix} I_{1n} \\ I_{2n} \\ \vdots \\ \\ \end{bmatrix} \tag{1}$$

ξ was estimated to be $\sim$0.19 and $\sim$0.18 for the red and blue arms, respectively, from a calibration image of alternating '1's and '0's transmitted by the DMDs. $\overline{I_{:n}}$ is renormalized after this matrix multiplication. We show the effects of crosstalk reduction in Fig. S2.

To maximize energy efficiency, the final version of this system (with a custom CMOS chip that integrates detection with digital MAC computation) will not perform post-processing. We can use charge-sharing at the detector pixels to implement equation (1) with custom CMOS. Alternatively, we could simply reduce crosstalk by changing the system design. For example, we could choose to space the PEs further apart or shrink the active region of the detectors to improve the ratio of signal at the current pixel to noise from neighboring pixels. Another option is a dual-rail scheme, where two detectors on opposite corners of a MAC unit detect one bit: light is sent to the first detector for a '1' or to the second detector for a '0'. The difference in charge between these neighbors is more robust to error compared with the absolute charge, which may cross the fixed threshold with sufficient crosstalk, but not reach a higher charge than its neighbors.

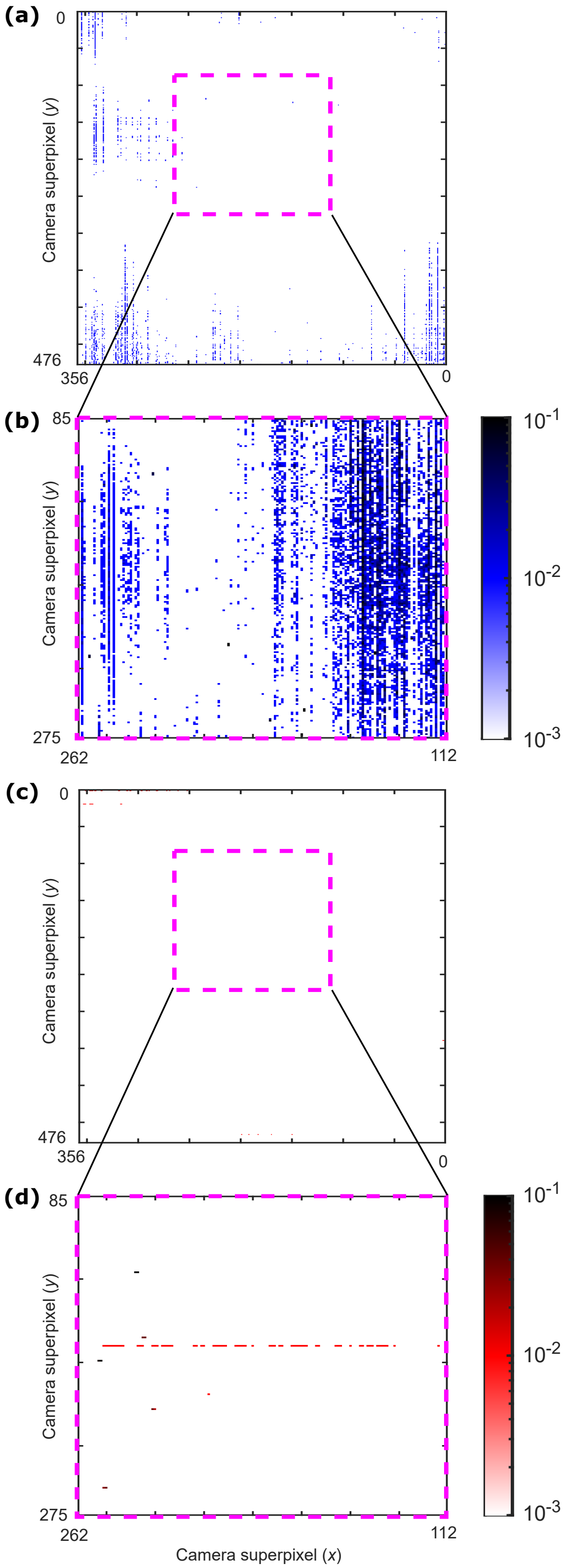

Figure S1. Bit error rates in proof-of-concept experiment. (a) Blue channel: errors when random vector of '0's and '1's displayed on DMD (single shot). Blue: incorrectly transmitted bit; white: correct. (b) Region of interest selected for experiment. Error in blue channel averaged over 100 frames (different vectors displayed on DMD at each frame). (c)-(d) same as (a)-(b), but for red channel.

Supplementary Note 3: Training and test sets

In our proof-of-concept experiment, we performed inference on 500 images using a two-hidden-layer, fully-connected neural network, where each hidden layer had 100 activations. We used TensorFlow's built-in dataset importer to download the first 500 images in the test set of the MNIST handwritten digit dataset[1], as downloaded from the TensorFlow 2 Keras database. Relevent code can be found in the GitHub repository for user Alexander Sludds (alexsludds):
https://github.com/alexsludds/Digital-Optical-Neural-Network-Code

The model's weights were pre-trained on an NVIDIA K40 GPU using the entire MNIST training set. Categorical cross-entropy was used as a loss function. Dropout regularized the model's weights in each layer to prevent overfitting. Input images were downsized from 49×49 to 7×7 using bilinear interpolation.

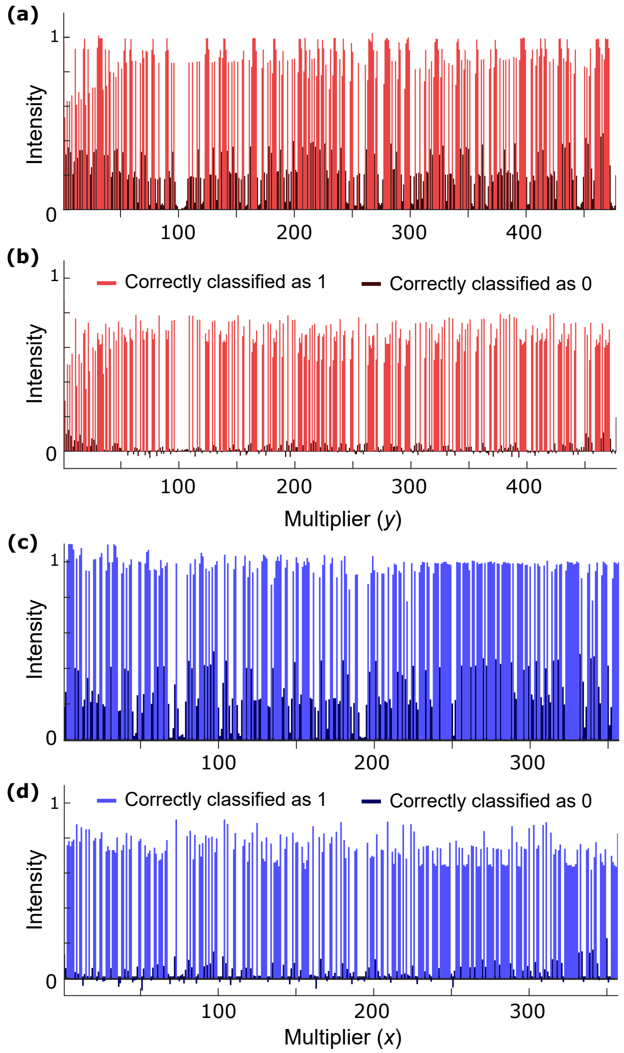

Figure S2. One line of receiver image after background subtraction and normalization, with random vectors of '1's and '0's displayed on DMDs. (a) Column 100 in red channel (same as Fig. 4b in main text). (b) Same as (a), after crosstalk correction. (c) Row 100 in blue channel. (d) Same as (c), after crosstalk correction.

Supplementary Note 4: Electronic interconnect switching energy in 0 to 1 transitions

The dynamic switching energy of CMOS devices is the amount of energy required to charge the output capacitance of a CMOS gate. Energy is only consumed in CMOS inverters for low-to-high transitions on the outputs of these gates. Consider the toy circuit model shown in Fig. S3. On the left is a CMOS inverter, and on the right are a low-to-high and high-to-low transitions, respectively. In the low-to-high transition, the PMOS has to switch closed, shorting the output to the supply rail by charging the load capacitance. In the high-to-low transition, the NMOS already has a sufficient drain-to-source voltage from the load

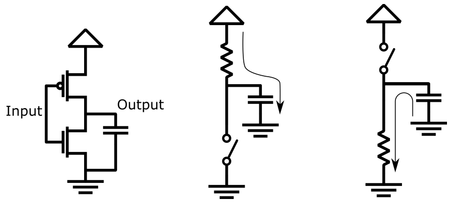

Figure S3. A demonstration of where dynamic energy consumption goes during switching of a CMOS inverter. The circuit, shown left, consists of a stacked NMOS and PMOS device. During an output low to high transition, shown center, charge is deposited on the lumped output capacitance. During an output low to high transition, shown right, that charge from the lumped output capacitance is discharged through the NMOS into ground.

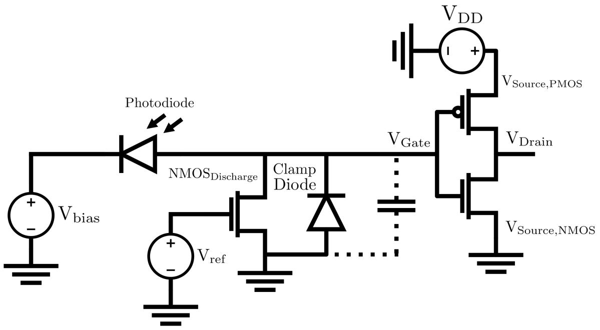

Figure S4. A proposed circuit for resetting the receiver lumped capacitor model.

capacitance charge, so it can discharge the output without consuming any power from the supply. To summarize, in an output which switches from low to high and back to low again, the PMOS initially turns on, taking CV_{DD}^2 energy from the supply, then the NMOS will turn on, discharging $\frac{1}{2}CV_{DD}^2$ from the charged load capacitor (the other $\frac{1}{2}CV_{DD}^2$ is dissipated as heat in the resistive load).

Supplementary Note 5: Resetting a 'receiverless' circuit

There are several circuit methods by which the accumulated charge on the input capacitor can be reset. In the method shown in Fig. S4, we place the NMOS device $\text{NMOS}_{\text{Discharge}}$ between the photodetector and ground and drive the gate with an external reference voltage V_{ref}. The benefit of this solution is that it consumes no dynamic energy when there is no optical input power. However, it has the tradeoff that it requires additional area on chip and, because it is ratioed logic, requires careful design to ensure functionality. The width of $\text{NMOS}_{\text{Discharge}}$ is set such that the accumulated charge on the capacitor generates a voltage high enough to overcome the input threshold of the load (modeled here as a CMOS inverter), but not so small that it cannot dissipate the charge quickly in a single clock cycle. One problem that arises from receiverless photodetection is that a constant steam of '1's coming into a photodetector without a strong enough $\text{NMOS}_{\text{Discharge}}$ fill causes additional charge to slowly build on the load capacitance. To compensate, we propose a P-N junction diode (Clamp Diode).

Supplementary Note 6: Electronic repeaters

A naive implementation of a repeater is a double inverter. The energy required is $C_{\text{T}}V_{\text{DD}}^2$, since in any transition, one inverter must be making a low-to-high transition and the other a high-to-low transition. As a result, in any 'flip' of a repeater, one inverter does not consume energy. Using the values in Table 2 of the main text, the cost of a repeater is .06 fJ/bit for an output low-to-high transition. Therefore, even in the worst-case scenario where we place a repeater between every multiplier in an array of abutted 8-bit MAC units, the inter-multiplier interconnect energy cost is larger than that of the repeater.

Supplementary Note 7: Shot and thermal noise

In a hypothetical crosstalk-free DONN, the remaining noise sources are thermal (Johnson) and shot noise. To gain insight into whether they would affect classification accuracy, we estimate the ensuing bit error rates (BERs). The detector registers a '1'

when $q \geq q_{\mathrm{D}}$ photoelectrons are generated, and a '0' when $q < q_{\mathrm{D}}$, where we assume the threshold charge is set by $q_{\mathrm{D}} = n_{\mathrm{p}}/2$ electrons. Fig. S5 illustrates the probability distributions of the number of photoelectrons, as well as the probabilities that a '0' is received when a '1' is sent (BER_1), and vice-versa (BER_0).

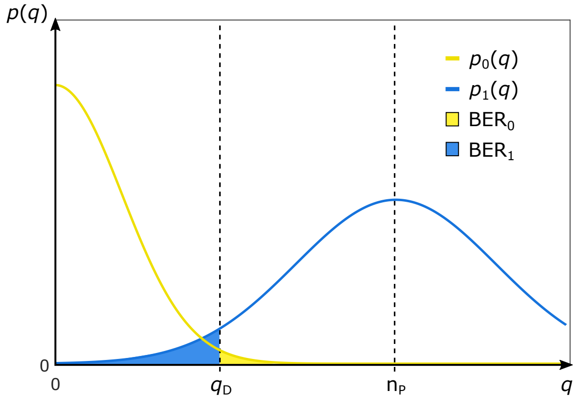

Figure S5. Schematic representation of probability density function of received charge (curves) and bit error rate (shaded region) - not to scale.

In a receiverless photodetector scheme, thermal noise can be approximated as 'kT/C' noise[2], with:

$$\sigma_{\mathrm{V}} = \sqrt{k_B T / (C_{\mathrm{det}} + C_{\mathrm{T}})} \tag{2}$$

where σ_{V} is the standard deviation of voltage, k_B is the Boltzmann constant, T is the temperature in Kelvin, C_{det} is the capacitance of the photodetector, and C_{T} is the capacitance of the inverter. The temperature depends on quality of heat sinking and proximity to hot spots; from the literature[3], we assume it is in the range $T \in [300-500]$. Using the values from Table 2 of the main text, we find $\sigma_{\mathrm{V}} \approx 5-6$ mV $\ll V_{DD}$. We can further verify whether thermal noise is likely to cause bit errors by approximating the probability distribution due to thermal noise, $p_{\mathrm{J}}(q)$, by a Gaussian:

$$p_{\mathrm{J}}(q) = \frac{1}{\sigma_{\mathrm{J}}\sqrt{2\pi}} e^{-\frac{q^2}{2\sigma_{\mathrm{J}}^2}} \tag{3}$$

with $\sigma_{\mathrm{J}} = \sqrt{k_B T (C_{\mathrm{det}} + C_{\mathrm{T}})}/e \approx 6-7$ electrons.

To first order, shot noise will not affect the transmission of '0's (BER_0) since the number of transmitted photons is $n_{\mathrm{p}} = 0$. Thus:

$$\mathrm{BER}_0 = \sum_{q=q_D}^{\infty} p_0(q) = \sum_{q=q_D}^{\infty} p_{\mathrm{J}}(q) = \sum_{q=q_D}^{\infty} \frac{1}{\sigma_{\mathrm{J}}\sqrt{2\pi}} e^{-\frac{q^2}{2\sigma_{\mathrm{J}}^2}} \tag{4}$$

$$\approx \frac{1}{2}\mathrm{erfc}\left(\frac{q_D}{\sqrt{2}\sigma_J}\right) \tag{5}$$

BER_0 for different $n_{\mathrm{p}} = 2q_{\mathrm{D}}$ are reported in Table 1.

We assume shot noise follows a Poissonian probability distribution:

$$p_{\mathrm{shot}}(q) = \frac{e^{-n_{\mathrm{p}}} (n_{\mathrm{p}})^q}{q!} \tag{6}$$

where n_{p} is the number of photons per detector per clock cycle.

For ease of computation with large n_{p}, we take the natural logarithm:

$$\ln\left(p_{\mathrm{shot}}(q)\right) = \ln\left(\frac{e^{-n_{\mathrm{p}}}\left(n_{\mathrm{p}}\right)^{q}}{q!}\right) \tag{7}$$

$$= \ln\left(e^{-n_{\mathrm{p}}}\right) + q\ln\left(n_{\mathrm{p}}\right) - \ln\left(q!\right) \tag{8}$$

$$= -n_{\mathrm{p}} + q\ln\left(n_{\mathrm{p}}\right) - \sum_{m=1}^{q}\ln\left(m\right) \tag{9}$$

$$\Downarrow \tag{10}$$

$$p_{\mathrm{shot}}(q) = \exp\left(-n_{\mathrm{p}} + q\ln\left(n_{\mathrm{p}}\right) - \sum_{m=1}^{q}\ln\left(m\right)\right) \tag{11}$$

BER_1 due to shot noise is therefore:

$$\mathrm{BER}_1^{\mathrm{shot}} = \sum_{q=1}^{q_{\mathrm{D}}-1} p_{\mathrm{shot}}(q) \tag{12}$$

Results of this computation for various n_{p} are shown in Table 1.

Table1. Expected values for BER_1 due to shot noise for different numbers of transmitted photons/bit

n_{p}	BER_0^*	$\mathrm{BER}_1^{\mathrm{shot}}$	$\mathrm{BER}_1^{\mathrm{total}}$
10	10^{-1}	10^{-2}	10^{-1}
100	$10^{-18} - 10^{-12}$	10^{-8}	$10^{-6} - 10^{-5}$
1000	small†	10^{-69}	$10^{-65} - 10^{-63}$

$^*\mathrm{BER}_0 = \mathrm{BER}_1^{\mathrm{thermal}}$
†Too small for MATLAB to compute.
Note: We report a range since thermal noise, and therefore BER, depends on quality of heat sinking.

Thermal noise will also contribute to BER_1; we convolve the probability distributions to find the total bit error rate:

$$\mathrm{BER}_1^{\mathrm{total}} = \sum_{q=1}^{q_{\mathrm{D}}-1} p_1(q) = \sum_{q=1}^{q_{\mathrm{D}}-1} p_{\mathrm{shot}}(q) \circledast p_{\mathrm{J}}(q) \tag{13}$$

From equation (5) in the main text, we find $n_{\mathrm{p}} \approx 1000$ photons/bit to generate a voltage swing of 0.8 V on the load capacitance; therefore, the expected BER is negligible, per Table 1.

Supplementary Note 8: Scalability of the DONN

In this section, we discuss the scalability of our proposed system. Free-space optical propagation is nearly lossless, so transmission distance will not be limiting in practice. It is true that there will be a limit to the power an individual source can produce ($\sim$100 mW for a single VCSEL[4]), which would appear to limit N's maximum value (N_{max}). In this case, using the values and equations from the main text, at a standard clock rate of 1 GHz:

$$100\ \mathrm{mW}/1\ \mathrm{GHz} = N_{\mathrm{max}} \times h\nu(C_{det} + C_T) \times V_{DD}/e \tag{14}$$

$$N_{\mathrm{max}} \approx 500{,}000 \tag{15}$$

We can then conservatively define a unit cell with a similar layout to Fig. 2c in the main text with N = 1,000 and B = 1,000. This unit cell can then be tiled (replicated) to increase the effective size of the array. Unit cells can be synchronized by optically transmitting values to them in the same manner that light sources are fanned out to receivers in the DONN. A tree-style structure distributes data from one central array to each of these unit cells where the branching points in the tree are a linear array of O-E-O devices which receive optical light, convert to an electronic signal and re-emit an amplified signal. Such a device could be implemented by connecting a receiverless photodetector to a CMOS repeater to a VCSEL. These devices can add

additional energy overhead, requiring an external power source to enable the creation of this new strong light signal. However, because higher levels of the tree are fanning out to successively many more roots, the cost of these devices can be substantially amortized (by a factor of roughly 1000^P, where P is the level of the tree). Thus, they do not add significant power overhead. In terms of fabrication of the unit cell, large, densely packed electronic multiplier arrays are already in use today, for example in the Google TPU[5].

Supplementary Note 9: Potential latency reduction from DONN architecture

There are several components which could limit the speed of operation of the DONN, namely the modulated sources, photodetectors, and receiver electronics.

Modulated sources

The bandwidth of a directly-driven incoherent light source such as a nanoLED can achieve bandwidths in excess of 10 GHz, though the current energy efficiency of these devices requires improvement[6]. Coherent sources, such as VCSELs driven by an electronic source, can achieve bandwidths exceeding 10 GHz[7].

Photodetector bandwidth

The bandwidth of a receiverless photodetector can be limited by one of two factors: the time for carrier removal or RC time constants at the detector. Well-designed photodetectors confine photocarriers between electrical contacts, which allows all carriers to be extracted by carrier drift rather than carrier diffusion, achieving bandwidths close to 100 GHz[8]. The RC time constant of tightly-integrated photodetectors (micrometer-scale wires) is orders of magnitude smaller than the carrier removal time.

Receiver electronics

A final factor which can limit the bandwidth of the system is the speed of driver and receiver electronics. Most modern high-throughput electronic systems have a clock speed limited by thermal constraints[9] of $\sim 10^6$ W/m^2. Considering an 8-bit MAC unit of energy $E_{\text{MAC}} = 25$ fJ/MAC and area 50 μm^2, the thermal limitation on operating speed for densely packed MAC logic with full utilization is $\sim$2 GHz. One benefit of the DONN architecture is the ability to increase distance between MAC units to overcome thermal constraints with no additional cost in data transfer. Increasing the pitch of the devices by 3.3$\times$ decreases the thermal density by 10$\times$, which allows for operation at up to 20 GHz.

References

1. LeCun, Y., Cortes, C. & Burges, C. J. C. MNIST handwritten digit database http://yann.lecun.com/exdb/mnist/. (1998) .

2. Miller, D. A. B. Attojoule optoelectronics for low-energy information processing and communications. *J. Light. Technol.* **35**, 346–396, DOI: 10.1109/JLT.2017.2647779 (2017).

3. Shrivastava, M. *et al.* Physical insight toward heat transport and an improved electrothermal modeling framework for FinFET architectures. *IEEE Transactions on Electron Devices* **59**, 1353–1363, DOI: 10.1109/TED.2012.2188296 (2012).

4. Peters, F. *et al.* High-power vertical-cavity surface-emitting lasers. *Electron. Lett.* **29**, 200–201, DOI: 10.1049/el:19930134 (1993).

5. Jouppi, N. P. *et al.* In-datacenter performance analysis of a tensor processing unit. In *Proceedings of the 44th Annual International Symposium on Computer Architecture*, 1–12, DOI: 10.1145/3079856.3080246 (2017).

6. Shambat, G. *et al.* Ultrafast direct modulation of a single-mode photonic crystal nanocavity light-emitting diode. *Nat. communications* **2**, 1–6, DOI: 10.1038/ncomms1543 (2011).

7. Koyama, F. Recent advances of VCSEL photonics. *J. lightwave technology* **24**, 4502–4513, DOI: 10.1109/JLT.2006.886064 (2006).

8. Lischke, S. *et al.* High bandwidth, high responsivity waveguide-coupled germanium pin photodiode. *Opt. express* **23**, 27213–27220, DOI: 10.1364/OE.23.027213 (2015).

9. Sun, Y., Agostini, N. B., Dong, S. & Kaeli, D. Summarizing CPU and GPU design trends with product data (2019). https://arXiv.org/abs/1911.11313.
